# Kidney function, brain morphology and cognition in the elderly: sex differences in the Austrian Stroke Prevention Study

**DOI:** 10.18632/aging.203829

**Published:** 2022-01-13

**Authors:** Michael Kolland, Edith Hofer, Lukas Pirpamer, Daniela Eibl, Christian Enzinger, Alexander R. Rosenkranz, Reinhold Schmidt

**Affiliations:** 1 Department of Internal Medicine, Division of Nephrology, Medical University of Graz, Graz 8036, Styria, Austria; 2 Department of Neurology, Division of Neurogeriatrics, Medical University of Graz, Graz 8036, Styria, Austria; 3 Institute for Medical Informatics, Statistics and Documentation, Medical University of Graz, Graz 8036, Styria, Austria; 4 Department of Neurology, Division of General Neurology, Medical University Graz, Graz 8036, Styria, Austria; 5 Division of Neuroradiology, Interventional and Vascular Radiology, Department of Radiology, Medical University of Graz, Graz 8036, Styria, Austria

**Keywords:** kidney function, brain morphology, sex, aging

## Abstract

Impaired kidney function is associated with structural brain changes and cognitive dysfunction. In the aging kidney, hemodynamic and structural alterations reduce the glomerular filtration rate (eGFR). Little is known about differences between men and women regarding decline of kidney function and brain damage.

In this community-based study, we assessed associations between the eGFR, focal and diffuse brain abnormalities and cognitive functions. Sex-specific effects were analyzed by interaction terms eGFR x sex on brain structure and cognition. Interactive effects were assessed using mixed-models –stratified by sex.

Overall, 196 women and 129 men (median age 68 years and mean eGFR 73.8±14.9 ml/min/1.73m^2^) were included. Significant associations existed between eGFR and cortical volume (β: 1.53E-04; SE: 6.72E-05; p=0.023 for neocortex). Sex exerted a significant interactive effect. Only in men, eGFR related to cortical volumes of all lobes and of deep gray matter structures (p= 0.001 for total gray matter, p=0.0004 for neocortex). In the whole group eGFR was not associated with cognition, but men with lower eGFR performed worse on tests for executive function, which, after FDR correction, was not significant.

We conclude, that in community-dwelling middle-aged and elderly individuals, reduced eGFR relates to brain volume loss in men but not in women.

## INTRODUCTION

Kidneys and brain are irrigated by short, small perforating arterioles, which auto-regulate perfusion pressure to maintain a continuous and stable high blood flow [1]. Both organs are affected by similar vascular risk factors such as age, hypertension, diabetes mellitus and smoking [2]. Individuals with chronic kidney disease (CKD) present a plethora of small vessel disease related brain changes [1–4] with focal and diffuse structural and microstructural abnormalities [5–7]. These include lacunar strokes, white matter abnormalities and microbleeds [3, 8, 9]. Already in the 1980ies, studies described a higher incidence of brain atrophy in patients with CKD when compared to the general population [10–13]. Both focal and diffuse brain changes are presumed to cause cognitive impairment of various degree in patients with renal dysfunction [14]. There is evidence for a sex paradox in kidney disease [15]. While women experience a higher prevalence of CKD, men are more likely to suffer kidney failure and have higher mortality rates in predialysis CKD [16, 17]. Yet, so far, there is hardly any data on sex differences of brain damage in the wake of milder kidney dysfunction.

Here, we examined the associations between eGFR and brain MRI findings in a large cohort of elderly persons without a history of strokes or dementia. We first determined if eGFR in an older community-dwelling population relates to focal and diffuse structural and microstructural brain changes as well as cognitive functions, and second, if these associations, when present, are indeed influenced by sex.

## RESULTS

In total, 196 women and 129 men (median age 68 years; IQR: 55–73), with a mean eGFR of 73.8 ± 14.9 ml/min/1.73m^2^ were included in the study. Baseline demographics and risk factors are shown in Table 1. Cardiovascular risk factors were common. Arterial hypertension was frequent in both men and women, but diastolic blood pressure values were higher in men. Also, significantly more men were smokers and men experienced more years of education and consumed more alcoholic drinks. They had higher levels of hemoglobin, transferrin saturation, homocysteine and urea. By contrast, women had higher levels of HbA1c, HDL and cholesterol.

**Table 1:** Demographics, risk factors and metabolic panel in the study cohort and differences between men and women.

	**ALL N= 325**	**MEN N=129**	**WOMEN N= 196**	p*
**Age (Years)** *(median [IQR])*	68[55–73]	67[51–73]	68[58–73]	0.392
**EGFR (ml/min/1.73m^2^)** *(mean±SD)*	73.8±15.0	75.4±14.2	72.8±15.4	0.127
**Risk factors**				
**Arterial hypertension** *present (N, %)*	209(64.3)	91 (70.5)	118(60.2)	0.057
**Systolic blood pressure (mmHg)** *(mean±SD)*	137.9 ± 21.1	139.3±19.1	136.9±22.3	0.318
**Diastolic blood pressure (mmHg)** *(mean±SD)*	86.6±9.2	88.3±9.5	85.5±8.8	**0.008**
**Diabetes mellitus** *present (N, %)*	32(9.8)	11(8.5)	21(10.7)	0.517
**Hypercholesterinemia** *present (N, %)*	240(73.8)	87(67.4)	153(78.1)	**0.033**
**BMI (kg/m^2)** *(mean±SD)*	26.4±4.5	26.9±3.7	26.2±4.9	0.132
**Years of education (Years)** *(Median [IQR])*	10 [10–13]	13 [10–18]	10 [9–13]	**<0.001**
**Alcohol consumption** *present (N, %)*	184(56.6)	96(74.4)	88(44.9)	**<0.001**
**Smoking status** *present (N, %)*	152(46.7)	77(59.7)	75(38.3)	**<0.001**
**Cardiac disease (History of CAD or AF)** *present (N, %)*	27(8.3)	6(4.6)	21(10.7)	0.053
**Metabolic panel**				
**HbA1c (mg/dL)** *(Median [IQR])*	5.5 [5.3 – 5.8]	5.5 [5.3 – 5.7]	5.6 [5.4 – 5.8]	**0.035**
**Cholesterol level (mg/dL)** *(mean±SD)*	208.7±40.1	198.42±38.58	215.43±39.67	**<0.001**
**HDL (mg/dL)** *(mean±SD)*	68.2±19.6	58.7±15.1	74.5±19.8	**<0.001**
**LDL level (mg/dL)** *(mean±SD)*	119.6±33.0	118.1±30.2	120.5±34.7	0.524
**Hemoglobin level (g/dL)** *(mean±SD)*	14.0±1.2	14.7±1.1	13.5±1.0	**<0.001**
**Transferrin saturation (%)** *(median [IQR])*	30.0 [34.0 – 37.0]	32.0 [25.0 – 40.0]	29.0 [23.3 – 35.0]	**0.002**
**Homocysteine (μmol/l)** *(Median [IQR])*	12.5 [10.6–14.6]	13.4 [11.8–15.5]	11.9 [10.3–13.9]	**<0.001**
**Urea (mg/dL)** *(median [IQR])*	33.0 [26.5 – 39.0]	34.0 [28.0 – 40.0]	31.0 [25.3 – 38.8]	**0.049**

*p-value for comparison between men and women obtained from Chi2 Test or Fisher’s exact test for binary variables and from t-test in case of normal distribution and Mann Whitney U Test in case of non-normal distribution of continuous variables.

IQR, interquartile range; SD, Standard Deviation; EGFR, estimated glomerular filtration rate; BMI, body mass index; CAD, Coronary artery disease; AF, Atrial fibrillation.

MRI findings were comparable between men and women, except for larger normalized hippocampal volumes in women than in men (p<0.001) and higher white matter hyperintensities volumes in men than in women (Table 2).

**Table 2:** Baseline MRI findings in the study cohort and differences between men and women.

**MRI**	**ALL** **N= 325**	**MEN** **N=129**	**WOMEN** **N= 196**	p*
**WMH volume~** *(median [IQR])*	0.0027 [0.0015-0.0052]	0.0046 [0.0014 – 0.0047]	0.0031 [0.0016 – 0.0059]	**0.049**
**Lacunes** *present (N, %)*	30(9.3%)	12(9.3%)	18(9.2%)	0.977
**Infarcts** *present (N, %)*	12(3.7%)	5(3.9%)	7(3.6%)	1.000
**Microbleeds** *present (N, %)*	27(8.5%)	13(10.2%)	14(7.4%)	0.370
**PSMD** (mm^2^/sec) *(N=236; mean±SD)*	0.00030±0.00005	0.00030±0.00005	0.00029±0.00005	0.144
**Total Gray Volume ~** *(mean±SD)*	0.3855±0.0220	0.3835±0.0246	0.3868±0.0199	0.174
**Cortical Volume ~** *(mean±SD)*	0.2731±0.0175	0.2721±0.0201	0.2738±0.0155	0.394
**Frontal Lobe Volume ~** *(mean±SD)*	0.1004±0.0075	0.0996±0.0084	0.1008±0.0070	0.160
**Parietal Lobe Volume~** *(mean±SD)*	0.0625±0.0049	0.0622±0.0052	0.0627±0.0048	0.410
**Temporal Lobe Volume~** *(mean±SD)*	0.0650±0.0043	0.0651±0.0051	0.0650±0.0039	0.858
**Occipital Lobe Volume~** *(mean±SD)*	0.0288±0.0026	0.0287±0.00287	0.0288±0.0025	0.741
**Hippocampus Volume~** *(mean±SD)*	0.0028±0.0002	0.0027±0.0002	0.0028±0.0002	**0.0007**
**Deep Gray Matter ~** *(mean±SD)*	0.0440±0.0027	0.0438±0.0026	0.0442±0.0027	0.218

*p-value for comparison between men and women obtained from Chi2 Test or Fisher’s exact test for binary variables and from t-test in case of normal distribution and Mann Whitney U Test in case of non-normal distribution of continuous variables.

IQR, interquartile range; SD, Standard Deviation; MRI, Magnetic Resonance Imaging; WMH, white matter hyperintensities; PSMD, peak width of skeletonized mean diffusivity.

~volumes (mm^3^) normalized for total intracranial volume.

Table 3 demonstrates the associations between eGFR and MRI findings in the total cohort. After adjustment for possible confounders and correction for multiple testing, there were no significant associations between eGFR and markers of cerebral small vessel disease including volumes of white matter hyperintensities (WMH) and peak width of skeletonized mean diffusivity (PSMD). Direct associations existed between eGFR and the volumes of the total neocortex and the cortical volumes of the parietal and occipital lobe (Table 3). EGFR was not related to cognitive functions including memory (β: -0.0035; SE: 0.0035, p=0.32), executive function (β: 0.0026, SE: 0.0023, p=0.25) and visuo-practical skills (β: -0.0008, SE: 0.0032, p=0.80) in the whole group.

**Table 3:** Associations between eGFR and structural- and microstructural MRI changes.

	**ALL N=325**		
	**β**	**SE**	p*
**PSMD~**	-3.09E-07	2.14E-07	0.149
WMH load**	3.33E-03	4.32E-03	0.441
**Total Gray Matter**	1.29E-04	8.41E-05	0.126
**Neocortex**	1.53E-04	6.72E-05	**0.023**
**Frontal Lobe**	5.09E-05	2.92E-05	0.082
**Parietal Lobe**	4.99E-05	1.93E-05	**0.010^#^**
**Temporal Lobe**	1.03E-05	1.81E-05	0.570
**Occipital Lobe**	2.22E-05	1.12E-05	**0.046**
**Deep Gray Matter**	1.39E-05	1.11E-05	0.212

Mixed model analyses determining the association between eGFR and MRI adjusted for age, education, smoking status, alcohol, hypertension, diabetes, hypercholesterolemia, HDL, cardiac diseases, diastolic BP, homocysteine, hemoglobin, transferrin saturation and family structure.

*p-value not corrected for multiple testing.

^#^p-value significant after false discovery rate (FDR) correction for multiple testing.

For PSMD (Peak width of Skeletonized Mean Diffusivity): N(Total)=236, N(Men)=92, N(women)=144.** WMH load was natural logarithm transformed, as the variable is not normally distributed.

eGFR, estimated glomerular filtration rate, Magnetic Resonance Imaging; β, regression coefficient; SE, standard error of the regression coefficient.

Table 4 shows the interaction terms eGFR x sex on brain volume. The interactions were significant for total gray matter, the neocortex and for volumes of the frontal-, and temporal lobe (Table 4).

**Table 4:** Interaction analysis for eGFR and sex on total and lobar brain volume.

	**β**	**SE**	p*
**Total Gray Matter**	-3.90E-04	1.48E-04	**0.008^#^**
**Neocortex**	-3.33E-04	1.18E-04	**0.005^#^**
**Frontal Lobe**	-1.30E-04	5.15E-05	**0.011^#^**
**Parietal Lobe**	-4.91E-05	3.43E-05	0.152
**Temporal Lobe**	-8.31E-05	3.19E-05	**0.009^#^**
**Occipital Lobe**	-3.23E-05	1.98E-05	0.103
**Deep Gray Matter**	-3.68E-05	1.97E-05	0.061

Interaction analysis model determining interaction between eGFR, MRI and sex adjusted for age, education, smoking status, alcohol, hypertension, diabetes, hypercholesterolemia, HDL, cardiac diseases, diastolic BP, homocysteine, hemoglobin and transferrin saturation. β, regression coefficient; SE, standard error of the regression coefficient.

*p-value not corrected for multiple testing.

^#^p-value significant after false discovery rate (FDR) correction for multiple testing.

Associations were only significant in men but not in women (Table 5).

**Table 5:** Association between eGFR and total and lobar brain volume.

	**MEN N=129**				**WOMEN N=196**		
	**β**	**SE**	p*		**β**	**SE**	p*
**Total Gray Matter**	5.00E-04	1.54E-04	**0.001^#^**		-5.43E-06	1.03E-04	0.958
**Neocortex**	4.42E-04	1.25E-04	**0.0004^#^**		3.07E-05	7.98E-05	0.701
**Frontal Lobe**	1.87E-04	5.49E-05	**0.0007^#^**		2.69E-07	3.46E-05	0.994
**Parietal Lobe**	9.24E-05	3.32E-05	**0.005^#^**		3.24E-05	2.49E-05	0.194
**Temporal Lobe**	7.11E-05	3.45E-05	**0.039**		-2.47E-05	2.15E-05	0.251
**Occipital Lobe**	5.01E-05	2.10E-05	**0.017**		1.29E-05	1.36E-05	0.344
**Deep Gray Matter**	5.10E-05	1.89E-05	**0.007^#^**		-6.01E-06	1.47E-05	0.682

Mixed model analyses determining the association between eGFR and MRI changes adjusted for age, education, smoking status, alcohol, hypertension, diabetes, hypercholesterolemia, HDL, cardiac diseases, diastolic BP, homocysteine, hemoglobin, transferrin saturation and family structure.

*p-value not corrected for multiple testing.

^#^p-value significant after false discovery rate (FDR) correction for multiple testing.

**Figure 1:**
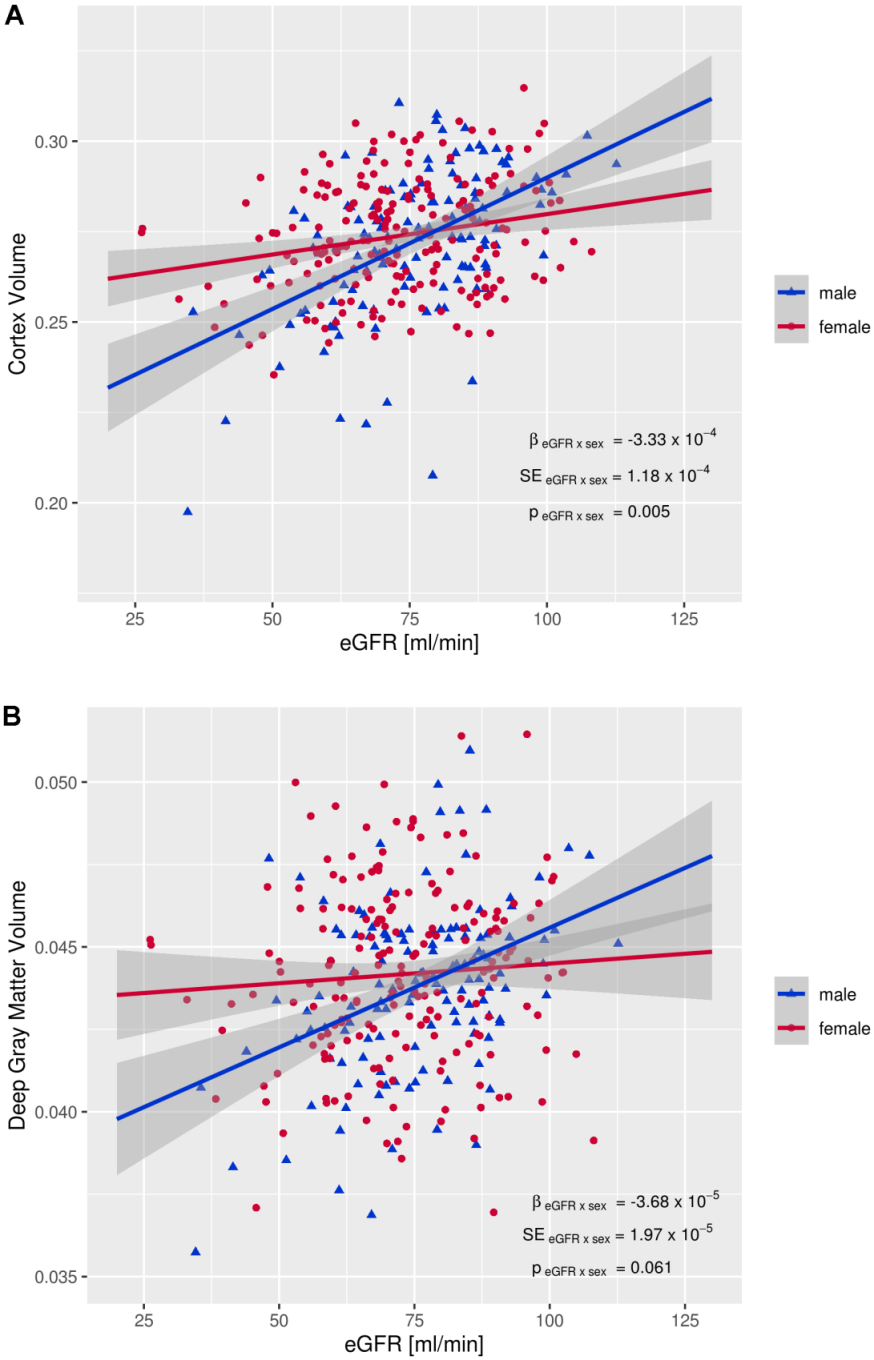
**Interaction between eGFR and sex on brain volumes.** (**A**) Interaction between eGFR and cortex volume, (**B**) Interaction between eGFR and deep gray matter volume. EGFR: estimated glomerular filtration rate, β _eGFR x sex_: regression coefficient of the eGFR x sex interaction term, SE _eGFR x sex_: standard error of the eGFR x sex interaction term, p _eGFR x sex_: p-value of eGFR x sex interaction term Brain volumes (mm^3^) are normalized for total intracranial volume.

Lower eGFR in men related to smaller brain volume in both the neocortex and deep cortical structures (Table 5). Figure 1A, 1B demonstrate scatterplots of the association between eGFR and neocortical as well as deep gray matter volumes. The associations were linear in both sexes with a substantially steeper slope in men compared to women.

As can be seen in Table 6, after adjustment for possible confounders, men with lower eGFR performed significantly worse on tests for executive function, but the association was no longer significant when correcting for multiple testing. The association was not mediated by total or lobar cortical volumes (Supplementary Table 1).

**Table 6:** Sex specific associations between eGFR and cognitive test performance.

	**MEN N=129**				**WOMEN N=196**		
	**β**	**SE**	p*		**β**	**SE**	p*
**Memory**	-0.0037	0.0070	0.600		-0.0037	0.0042	0.380
**Executive function**	0.0090	0.0040	**0.026**		-0.0019	0.0028	0.500
**Visuo-practical skills**	0.0041	0.0051	0.420		-0.0040	0.0041	0.300

Mixed model analyses determining the association between eGFR and memory, executive function and visuopractical skills adjusted for age, education, smoking status, alcohol, hypertension, diabetes, hypercholesterolemia, HDL, cardiac diseases, diastolic BP, homocysteine, hemoglobin, transferrin saturation and family structure. *p-value not corrected for multiple testing. After false discovery rate (FDR) correction for multiple testing none of the p-values were significant.

eGFR, estimated glomerular filtration rate; β, regression coefficient; SE, standard error of the regression coefficient.

## DISCUSSION

In this cross-sectional analysis of 325 community-dwelling people from the Austrian Stroke Prevention Family Study (ASPS-Fam), there were no significant associations between eGFR and vascular brain lesions, but eGFR was directly related to normalized brain volume. The association affected the cortex and sex exerted an interactive effect. Decreasing eGFR related to smaller gray matter volume in men but not in women. In men, significant direct relationships were seen for all lobes and for deep gray matter structures. We also examined the relationship between eGFR and cognitive functioning and found no significant association in the whole group. Nonetheless, in men but not in women, reductions in eGFR were related to executive dysfunction after correction for confounding factors. This relationship was not mediated by global or lobar atrophy.

These observations were made even though 88% of our male study participants had eGFR values above 60 ml/min/1.73m^2^, representing a normal or only mild reduced kidney function.

Previous studies reported correlations between kidney function and brain atrophy mainly in patients with advanced CKD [11, 18, 19], thus, our study extends this finding to patients with only mildly impaired renal function. Recently, it has been shown that cystatin C was associated with cognitive performance, brain imaging pathology and decline to dementia in 90+-year-olds with a mean eGFR of 39ml/min/1.73 m^2^ [20]. Kidney function decreases by approximately 6ml/min/1.73m^2^ per decade [21]. A faster decline in men compared to women has been reported (0.55 ± 1.47 ml/min/1.73 m^2^ vs. −0.33 ± 1.41 ml/min/1.73 m^2^ per year, respectively) [22]. A large meta-analysis and a recent analysis of the Chronic Renal Insufficiency Cohort (CRIC) also described a more rapid rate of progression and worse kidney function outcome in male patients with CKD [23, 24]. This suggests that women might have a higher tolerability against CKD. If this also applies to kidney-related end-organ damage is yet undetermined.

In the context of our study results it is important to note that sex differences have also been reported for the age dependent decline in the volume of cortical as well as subcortical gray matter, with faster progression in men [25]. Previously, sex specific effects of aging on cognition have also been reported [26]. Gur et al. described that men showed a stronger age-related decline in cognitive functions including attentional deficits compared to women [26]. Accordingly, we cannot exclude that the relationship between loss of kidney function, brain volume reduction and decline in cognition seen in our study was not causal, but rather an age-related process which develops in parallel in both organs and is more rapid in men than in women. It is of note that the association between reduced eGFR and worse performance on tests of executive functioning in men was not mediated by global or lobar brain atrophy.

At this point the cause for sex differences in age-related reduction of eGFR, brain volume loss and cognitive dysfunction is not fully determined.

Conceivably women may experience a slower decline in renal function, brain volume and cognitive functions with age, because of estrogen’s nephro-and neuroprotective properties [27, 28]. Vice versa, a faster decline in eGFR, cerebral gray matter volume and of cognitive impairment may be due to the unhealthier lifestyle of men [16]. Vascular risk factors are known to accelerate the age-related damage in both organs. As expected, in our study, men indeed had more prevalent risk factors than women, and although our statistical analyses adjusted for these differences between men and women, residual confounding is still possible. The third explanation is that, despite the modest and most likely age-related reduction of renal function, systemic factors which act differentially between sexes are responsible for the increased brain volume loss and executive function deficits in our male participants. Little is known about sex-dependent differential expression of harmful factors in the wake of kidney dysfunction. Although speculative, one of many examples might be the increased level of circulating angiotensin II in men as compared to women in the presence of kidney dysfunction considering that angiotensin II has been reported to induce proinflammatory effects in the brain [29].

Our study has several strengths and limitations. Strengths are the well-characterized large cohort of 325 patients, extensive neuropsychological testing and quantitatively assessed MR imaging. Limitations include the cross-sectional design and lack of early indicators for kidney impairment such as FGF23 and lack of measurement of proteinuria.

The clinical relevance of our study findings is yet unclear. Longitudinal assessment is needed to determine the mid-and long-term outcome of study participants with respect to structural and functional brain changes. By all means our study findings are likely to augment the interest of future research on sex-dependency of cerebral abnormalities associated with renal dysfunction.

## MATERIALS AND METHODS

### Study subjects

Study participants are from the Austrian Stroke Prevention Family Study (ASPS-Fam), a prospective single-center community-based cross-sectional study designed to assess the cerebral effects of vascular risk factors in the healthy elderly population of the City of Graz, Austria. The Austrian Stroke Prevention Family Study represents an extension of the Austrian Stroke Prevention Study (ASPS), which was established in 1991 [30]. Between 2006 and 2013, study participants of the ASPS and their first-degree relatives were invited to enter the ASPS-Fam [31, 32]. The prospective study was approved by the local medical ethics committee of the Medical University Graz and signed written informed consent was obtained from all study participants. Individuals were excluded from the study, if they had had a history of neuropsychiatric disease, including previous cerebrovascular attacks and dementia or abnormal findings in the neurologic examination. Structured clinical interviews and a physical and neurologic examinations were done by a board-certified neurologist. All participants were fully ambulatory and functionally independent subjects and they had no signs of heart failure and no visual impairment that might have affected neuropsychological testing. The entire cohort underwent an extended diagnostic work-up including clinical history, blood tests, cognitive testing, and a thorough vascular risk factor assessment. Four hundred and nineteen subjects were included and those 325 individuals with complete laboratory, brain Magnetic Resonance Imaging (MRI), cognition and risk factor data comprised the current cohort.

Kidney function was determined using the CKD-EPI (CKD Epidemiology Cooperation) equation based on isotope-dilution mass spectrometry-validated serum creatinine concentrations. Assessment of vascular risk factors included arterial hypertension, diabetes mellitus, body mass index, hypercholesterolemia, cardiac disease, history of smoking, alcohol consumption and was determined based on history and measurements at the examination as previously described [30].

Briefly, arterial hypertension was considered as a medical history of hypertension with repeated blood pressure values higher than 140/90 mmHg, medical treatment for hypertension or readings at the examination exceeding blood pressure values of 140/90 mmHg (ESH/ESC Guidelines 2013). Diabetes mellitus was coded present if an individual was treated for diabetes at the time of examination or if the fasting blood glucose level at the examination exceeded 126 mg/dl. BMI was defined according to WHO definition [33]. A lipid status was determined with standardized measurements for each study participant after 12-hour fasting to assess the presence of hyperlipidemia. Hypercholesterolemia was defined as history of hypercholesterolemia, medical treatment for hypercholesterolemia, total cholesterol exceeding 200 mg/dl or HDL cholesterol exceeding 130 mg/dl. Cardiac disease included a history of coronary heart disease or atrial fibrillation. Study participants were asked whether they were previous and/or current smokers or habitual daily alcohol drinkers. Moreover, hemoglobin level, transferrin saturation and homocysteine concentrations, were obtained from serum measurements.

### Magnetic resonance imaging (MRI)

MRI of the brain was performed at a 3T whole body scanner (TimTrio; Siemens Healthcare, Erlangen, Germany) and included conventional imaging and diffusion weighted imaging (DWI). The conventional protocol included an axial Fluid Attenuated Inversion Recovery (FLAIR) sequence (TR = 10000ms, TE = 69ms, inversion time = 2500ms, number of slices = 40, slice thickness = 3mm, in-plane resolution = 0.86mm x 0.86mm) and a high resolution T1 weighted 3D magnetization-prepared rapid acquisition gradient-echo (MPRAGE) sequence with whole brain coverage (TR = 1900ms, TE = 2.19ms, inversion time = 900ms, flip angle = 9°, isotropic resolution of 1mm).

Vascular lesions including white matter hyperintensities (WMH), silent non-lacunar and lacunar infarcts were assessed on FLAIR images by a blinded expert. WMH were outlined using a custom written IDL program (Exelis Visual Information Solutions, USA). Lesion areas were segmented semi-automatically by a trained rater as previously described [34]. Total lesion volume (cubic millimeter) was calculated using fslstats (part of FSL, freely available at https://FSL.fmrib.ox.ac.uk) and normalized by each subject’s head size. Due to a skewed distribution, the lesion load was logarithmically transformed. Lacunes were defined as focal lesions involving the basal ganglia, internal capsule, thalamus, brainstem, or the white matter, not exceeding a maximum diameter of 20 mm. We considered lesions with typical signal characteristics of infarcts following a typical vascular territory or located in a border zone between two vascular territories as non-lacunar infarcts.

To assess microstructural changes, we used the peak width of the skeletonized mean diffusivity (PSMD) (freely available at: http://www.psmd-marker.com/). This represents a recently established, robust, fully automated, and easy-to-implement marker for cerebral small vessel disease based on diffusion tensor imaging, white matter tract skeletonization (as implemented in FSL-TBSS) and histogram analysis. The PSMD-marker was performed in 236 subjects.

Regional volumetric assessments of cortical and deep GM structures were performed fully automated using the structural imaging stream “recon-all” from FreeSurfer (version 6.0; documented and freely available for download online http://surfer.nmr.mgh.harvard.edu). The processing includes segmentation of the subcortical white matter and deep GM volumetric structures and parcellation of the cerebral cortex into regions, based on gray and sulcal structures [35, 36]. For our analyses we considered the total GM volume consisting of neocortex and deep GM as well as lobar cortical (frontal, parietal, temporal and occipital) and hippocampal volume. All volumes were normalized for total intracranial volume.

### Neuropsychological testing

A test battery assessing memory, executive function and motor skills was applied as described previously [30]. These tests are widely used in the German-speaking area and were applied in the same order and under same laboratory conditions. Intermediate memory recall and learning ability was assessed by the “Bäumler’s Lern- und Gedächtnistest” (LGT-3), a highly demanding paper–pencil procedure consisting of six subtests. Three subtests (word and digit association tasks, and story recall) screen for verbal memory and two subtests (trail and design recall) screen for visuospatial memory. The sum of weighted scores from these subtests and of an image recognition paradigm result in a total learning and memory performance score. The stimulus sets of the word association task (German-Turkish word pairs), the story (facts about construction of a library), and design recall (core symbol and frame), and the recognition paradigm (objects) consist of 20 items each. A trail in an abstracted city map serves as the trail recall test. These sets of stimuli were presented to the person being tested for 1 minute. Two minutes were given for learning 13 items of the digit association task (three-digit telephone numbers and names of extension holders). During a learning phase, the six sets of stimuli are subsequently presented to the person being tested. The recall phase starts immediately thereafter and follows the same order. Delay between presentation and recall for a given subtest ranges between 7 and 11 minutes. Executive functions were tested by the Wisconsin card sorting test, part B of the trail making test [37], and digit span backwards, which is part of the Wechsler adult intelligence scale, revised [38]. Adhering to Milner’s criteria [39] the measures computed for the Wisconsin card sorting test were the categories “completed”, “perseverative errors”, and “total errors”. Motor skills were evaluated by the Purdue pegboard test.

To reduce floor and ceiling artifacts and other sources of measurement error, we used summary measures of cognitive function in the analyses rather than results of individual tests. We formed composite measures of the specific domains of cognitive function. Each summary measure was calculated by converting individual test scores to *z*-scores within a group and by computing the average of scores in each cognitive domain. Before the z-score conversion, individual test scores were reverse coded if necessary to ensure that higher scores reflect better cognitive function.

### Data analysis

Statistical analysis was performed using Stata SE 9, R software environment version 3.6.1 and IBM SPSS 25 Statistics software. Assumptions of normal distribution were tested with the Shapiro-Wilk test. Normally distributed variables are reported as mean ± standard deviation and non-normally distributed variables as median and interquartile range (IQR). The difference between men and women in demographics, risk factors, laboratory and MRI findings were calculated using chi-squared test or Fisher’s exact test for categorical variables, t-test or Mann-Whitney U test for normally or non-normally distributed variables, respectively. We performed linear mixed-effects model analyses to relate eGFR to MRI findings and to cognition. For each MRI finding and each cognitive domain, we calculated a model using eGFR as the independent variable and the MRI finding or cognitive domain as the dependent variable. Additionally, we performed interaction analyses for each MRI finding, including sex, eGFR and the sex x eGFR interaction term as independent variables and the MRI finding as the dependent variable, to determine if the association between eGFR and MRI findings was significantly different between men and women. Selection of covariates was based on evidence from previous literature [40]. We included age, education, vascular risk factors including arterial hypertension, diabetes mellitus, hypercholesterolemia, cardiac disease, smoking, alcohol consumption, as well as laboratory findings as covariates in the mixed models. To account for the relatedness of family members, a random effect was added to each model using a kinship matrix describing the family structure as implemented in the lmekin function of the R package coxme (https://cran.r-project.org/web/packages/coxme/vignettes/lmekin.pdf). Visual inspection of residual plots did not show deviations of mixed-effects model assumptions. Within each table, p-values obtained from the mixed models were corrected for multiple testing using the false discovery rate (FDR). P values < 0.05 were considered statistically significant.

The contribution of brain MRI findings on the association between eGFR and cognition was assessed by simple bootstrapped mediation models for estimating indirect effect sizes using the SPSS macro PROCESS v3.3. We applied the following definition: If the 95% confidence interval of the indirect effect did not contain 0, a significant mediation effect was deemed probable, whereas we assumed no mediation to be present if 0 was included in the 95% confidence interval.

### Data availability statement

The datasets generated and/or analysed during this study are not publicly available, but all of the individual participant data collected during the trial, after deidentification, Study Protocol, Statistical Analysis Plan, Informed Consent Form and Clinical Study Report, are available from the corresponding author on reasonable request. No end data after publication.

## Supplementary Material

Supplementary Table 1

## References

[1] Lau WL, Huisa BN, Fisher M. The Cerebrovascular-Chronic Kidney Disease Connection: Perspectives and Mechanisms. Transl Stroke Res. 2017; 8:67–76. 10.1007/s12975-016-0499-x 27628245 PMC5241336

[2] Khatri M, Wright CB, Nickolas TL, Yoshita M, Paik MC, Kranwinkel G, Sacco RL, DeCarli C. Chronic kidney disease is associated with white matter hyperintensity volume: the Northern Manhattan Study (NOMAS). Stroke. 2007; 38:3121–6. 10.1161/STROKEAHA.107.493593 17962588 PMC2948438

[3] Ikram MA, Vernooij MW, Hofman A, Niessen WJ, van der Lugt A, Breteler MM. Kidney function is related to cerebral small vessel disease. Stroke. 2008; 39:55–61. 10.1161/STROKEAHA.107.493494 18048865

[4] Vemuri P, Knopman DS, Jack CR Jr, Lundt ES, Weigand SD, Zuk SM, Thostenson KB, Reid RI, Kantarci K, Slinin Y, Lakshminarayan K, Davey CS, Murray A. Association of Kidney Function Biomarkers with Brain MRI Findings: The BRINK Study. J Alzheimers Dis. 2017; 55:1069–82. 10.3233/JAD-160834 27767995 PMC5621389

[5] Zhang R, Liu K, Yang L, Zhou T, Qian S, Li B, Peng Z, Li M, Sang S, Jiang Q, Sun G. Reduced white matter integrity and cognitive deficits in maintenance hemodialysis ESRD patients: a diffusion-tensor study. Eur Radiol. 2015; 25:661–8. 10.1007/s00330-014-3466-5 25326436

[6] Eldehni MT, Odudu A, Mcintyre CW. Brain white matter microstructure in end-stage kidney disease, cognitive impairment, and circulatory stress. Hemodial Int. 2019; 23:356–65. 10.1111/hdi.12754 30920718

[7] Saini S, Kumar V, Koteshwara P. Role of Diffusion Tensor Imaging in renal parenchymal changes. Indian J Radiol Imaging. 2018; 28:175–81. 10.4103/ijri.IJRI_128_17 30050240 PMC6038220

[8] Shima H, Ishimura E, Naganuma T, Yamazaki T, Kobayashi I, Shidara K, Mori K, Takemoto Y, Shoji T, Inaba M, Okamura M, Nakatani T, Nishizawa Y. Cerebral microbleeds in predialysis patients with chronic kidney disease. Nephrol Dial Transplant. 2010; 25:1554–9. 10.1093/ndt/gfp694 20037183

[9] Wada M, Nagasawa H, Iseki C, Takahashi Y, Sato H, Arawaka S, Kawanami T, Kurita K, Daimon M, Kato T. Cerebral small vessel disease and chronic kidney disease (CKD): results of a cross-sectional study in community-based Japanese elderly. J Neurol Sci. 2008; 272:36–42. 10.1016/j.jns.2008.04.029 18541269

[10] Passer JA. Cerebral atrophy in end-stage uremia. Proc Clin Dial Transplant Forum. 1977; 7:91–4. 616005

[11] Tsuruya K, Yoshida H, Haruyama N, Fujisaki K, Hirakata H, Kitazono T. Clinical Significance of Fronto-Temporal Gray Matter Atrophy in Executive Dysfunction in Patients with Chronic Kidney Disease: The VCOHP Study. PLoS One. 2015; 10:e0143706. 10.1371/journal.pone.0143706 26632813 PMC4669129

[12] Prohovnik I, Post J, Uribarri J, Lee H, Sandu O, Langhoff E. Cerebrovascular effects of hemodialysis in chronic kidney disease. J Cereb Blood Flow Metab. 2007; 27:1861–9. 10.1038/sj.jcbfm.9600478 17406658

[13] Fazekas G, Fazekas F, Schmidt R, Kapeller P, Offenbacher H, Krejs GJ. Brain MRI findings and cognitive impairment in patients undergoing chronic hemodialysis treatment. J Neurol Sci. 1995; 134:83–8. 10.1016/0022-510x(95)00226-7 8747848

[14] Bugnicourt JM, Godefroy O, Chillon JM, Choukroun G, Massy ZA. Cognitive disorders and dementia in CKD: the neglected kidney-brain axis. J Am Soc Nephrol. 2013; 24:353–63. 10.1681/ASN.2012050536 23291474

[15] Tomlinson LA, Clase CM. Sex and the Incidence and Prevalence of Kidney Disease. Clin J Am Soc Nephrol. 2019; 14:1557–9. 10.2215/CJN.11030919 31649072 PMC6832053

[16] Carrero JJ, Hecking M, Chesnaye NC, Jager KJ. Sex and gender disparities in the epidemiology and outcomes of chronic kidney disease. Nat Rev Nephrol. 2018; 14:151–64. 10.1038/nrneph.2017.181 29355169

[17] Stevens LA, Li S, Wang C, Huang C, Becker BN, Bomback AS, Brown WW, Burrows NR, Jurkovitz CT, McFarlane SI, Norris KC, Shlipak M, Whaley-Connell AT, et al. Prevalence of CKD and comorbid illness in elderly patients in the United States: results from the Kidney Early Evaluation Program (KEEP). Am J Kidney Dis. 2010; 55:S23–33. 10.1053/j.ajkd.2009.09.035 20172445 PMC4574484

[18] Meurs M, Roest AM, Groenewold NA, Franssen CF, Westerhuis R, Kloppenburg WD, Doornbos B, Beukema L, Lindmäe H, de Groot JC, van Tol MJ, de Jonge P. Gray matter volume and white matter lesions in chronic kidney disease: exploring the association with depressive symptoms. Gen Hosp Psychiatry. 2016; 40:18–24. 10.1016/j.genhosppsych.2016.02.005 27040607

[19] Yakushiji Y, Nanri Y, Hirotsu T, Nishihara M, Hara M, Nakajima J, Eriguchi M, Nishiyama M, Hara H, Node K. Marked cerebral atrophy is correlated with kidney dysfunction in nondisabled adults. Hypertens Res. 2010; 33:1232–7. 10.1038/hr.2010.171 20944639

[20] Lau WL, Fisher M, Greenia D, Floriolli D, Fletcher E, Singh B, Sajjadi SA, Corrada MM, Whittle C, Kawas C, Paganini-Hill A. Cystatin C, cognition, and brain MRI findings in 90+-year-olds. Neurobiol Aging. 2020; 93:78–84. 10.1016/j.neurobiolaging.2020.04.022 32473464 PMC7307913

[21] Denic A, Glassock RJ, Rule AD. Structural and Functional Changes With the Aging Kidney. Adv Chronic Kidney Dis. 2016; 23:19–28. 10.1053/j.ackd.2015.08.004 26709059 PMC4693148

[22] Halbesma N, Brantsma AH, Bakker SJ, Jansen DF, Stolk RP, De Zeeuw D, De Jong PE, Gansevoort RT, and PREVEND study group. Gender differences in predictors of the decline of renal function in the general population. Kidney Int. 2008; 74:505–12. 10.1038/ki.2008.200 18496511

[23] Ricardo AC, Yang W, Sha D, Appel LJ, Chen J, Krousel-Wood M, Manoharan A, Steigerwalt S, Wright J, Rahman M, Rosas SE, Saunders M, Sharma K, et al, and CRIC Investigators. Sex-Related Disparities in CKD Progression. J Am Soc Nephrol. 2019; 30:137–46. 10.1681/ASN.2018030296 30510134 PMC6317604

[24] Neugarten J, Acharya A, Silbiger SR. Effect of gender on the progression of nondiabetic renal disease: a meta-analysis. J Am Soc Nephrol. 2000; 11:319–29. 10.1681/ASN.V112319 10665939

[25] Király A, Szabó N, Tóth E, Csete G, Faragó P, Kocsis K, Must A, Vécsei L, Kincses ZT. Male brain ages faster: the age and gender dependence of subcortical volumes. Brain Imaging Behav. 2016; 10:901–10. 10.1007/s11682-015-9468-3 26572143

[26] Gur RE, Gur RC. Gender differences in aging: cognition, emotions, and neuroimaging studies. Dialogues Clin Neurosci. 2002; 4:197–210. 10.31887/DCNS.2002.4.2/rgur 22033483 PMC3181676

[27] Vegeto E, Benedusi V, Maggi A. Estrogen anti-inflammatory activity in brain: a therapeutic opportunity for menopause and neurodegenerative diseases. Front Neuroendocrinol. 2008; 29:507–19. 10.1016/j.yfrne.2008.04.001 18522863 PMC2630539

[28] Ahmed SB, Ramesh S. Sex hormones in women with kidney disease. Nephrol Dial Transplant. 2016; 31:1787–95. 10.1093/ndt/gfw084 27190328

[29] Biancardi VC, Stern JE. Compromised blood-brain barrier permeability: novel mechanism by which circulating angiotensin II signals to sympathoexcitatory centres during hypertension. J Physiol. 2016; 594:1591–600. 10.1113/JP271584 26580484 PMC4799983

[30] Schmidt R, Fazekas F, Kapeller P, Schmidt H, Hartung HP. MRI white matter hyperintensities: three-year follow-up of the Austrian Stroke Prevention Study. Neurology. 1999; 53:132–9. 10.1212/wnl.53.1.132 10408549

[31] Seiler S, Pirpamer L, Hofer E, Duering M, Jouvent E, Fazekas F, Mangin JF, Chabriat H, Dichgans M, Ropele S, Schmidt R. Magnetization transfer ratio relates to cognitive impairment in normal elderly. Front Aging Neurosci. 2014; 6:263. 10.3389/fnagi.2014.00263 25309438 PMC4174770

[32] Ghadery C, Pirpamer L, Hofer E, Langkammer C, Petrovic K, Loitfelder M, Schwingenschuh P, Seiler S, Duering M, Jouvent E, Schmidt H, Fazekas F, Mangin JF, et al. R2* mapping for brain iron: associations with cognition in normal aging. Neurobiol Aging. 2015; 36:925–32. 10.1016/j.neurobiolaging.2014.09.013 25443291

[33] Obesity: preventing and managing the global epidemic. Report of a WHO consultation. World Health Organ Tech Rep Ser. 2000; 894: i-xii, 1–253. 11234459

[34] Plummer DL. DispImage: A Display and Analysis Tool for Medical Images. Rivista di Neuroradiologia. 1992; 5:489–95. 10.1177/197140099200500413

[35] Desikan RS, Ségonne F, Fischl B, Quinn BT, Dickerson BC, Blacker D, Buckner RL, Dale AM, Maguire RP, Hyman BT, Albert MS, Killiany RJ. An automated labeling system for subdividing the human cerebral cortex on MRI scans into gyral based regions of interest. Neuroimage. 2006; 31:968–80. 10.1016/j.neuroimage.2006.01.021 16530430

[36] Fischl B, van der Kouwe A, Destrieux C, Halgren E, Ségonne F, Salat DH, Busa E, Seidman LJ, Goldstein J, Kennedy D, Caviness V, Makris N, Rosen B, Dale AM. Automatically parcellating the human cerebral cortex. Cereb Cortex. 2004; 14:11–22. 10.1093/cercor/bhg087 14654453

[37] Department USW. Army Individual Test Battery: Manual of directions and scoring. Washington. In: War Department AGsO, editor. Washington 1944.

[38] Tewes U. HAWIE-R, Hamburg-Wechsler Intelligenztest für Erwachsene, Revision 1991. Bern; Göttingen; Toronto; Seattle: Verlag Hans Huber; 1994 1991.

[39] Milner B. Effects of Different Brain Lesions on Card Sorting: The Role of the Frontal Lobes. JAMA Neurology. 1963; 9:90–100. 10.1001/archneur.1963.00460070100010

[40] Cox SR, Lyall DM, Ritchie SJ, Bastin ME, Harris MA, Buchanan CR, Fawns-Ritchie C, Barbu MC, de Nooij L, Reus LM, Alloza C, Shen X, Neilson E, et al. Associations between vascular risk factors and brain MRI indices in UK Biobank. Eur Heart J. 2019; 40:2290–300. 10.1093/eurheartj/ehz100 30854560 PMC6642726

