# Supplementary material for: Kidney function, brain morphology and cognition in the elderly: sex differences in the Austrian Stroke Prevention Study
Source: Aging (Albany NY). 2022 Jan 13;14(1):240–52. doi: 10.18632/aging.203829 (PMC8791200; doi:10.18632/aging.203829)
Supplement: Supplementary Table 1 [file aging-14-203829-s001.pdf]

SUPPLEMENTARY TABLE

Supplementary Table 1. Mediation analysis assessing the effect of structural and microstructural MRI changes on the relationship between eGFR and executive function in men (N=129).

Mediator	Indirect effect*	SE	Lower CI	Upper CI
Neocortex	0.0020	0.0018	-0.0011	0.0061
Frontal Lobe	0.0013	0.0014	-0.0012	0.0046
Parietal Lobe	0.0016	0.0015	-0.0006	0.0051
Temporal Lobe	0.0007	0.0010	-0.0008	0.0034
Occipital Lobe	0.0010	0.0013	-0.0010	0.0040

Mediation analysis adjusted for age, education, smoking status, alcohol, hypertension, diabetes, hypercholesterolemia, HDL, diastolic BP, Homocystein, Haemoglobin and Transferrin saturation.

*Indirect effect of eGFR on executive function. SE, standard error of the indirect effect; CI, bootstrapped confidence interval of the indirect effect; eGFR, estimated glomerular filtration rate.
